# She Doesn’t Even Go Here: The Role of Inflammatory Astrocytes in CNS Disorders

**DOI:** 10.3389/fncel.2021.704884

**Published:** 2021-09-03

**Authors:** Jacqueline Kelsey Reid, Hedwich Fardau Kuipers

**Affiliations:** ^1^Department of Clinical Neurosciences, Hotchkiss Brain Institute and Snyder Institute for Chronic Diseases, University of Calgary, Calgary, AB, Canada; ^2^Department of Cell Biology & Anatomy, Hotchkiss Brain Institute and Snyder Institute for Chronic Diseases, University of Calgary, Calgary, AB, Canada

**Keywords:** astrocytes, reactive astrogliosis, heterogeneity, neuroinflammation, immune mediators, CNS disorders

## Abstract

Astrocyte heterogeneity is a rapidly evolving field driven by innovative techniques. Inflammatory astrocytes, one of the first described subtypes of reactive astrocytes, are present in a variety of neurodegenerative diseases and may play a role in their pathogenesis. Moreover, genetic and therapeutic targeting of these astrocytes ameliorates disease in several models, providing support for advancing the development of astrocyte-specific disease modifying therapies. This review aims to explore the methods and challenges of identifying inflammatory astrocytes, the role these astrocytes play in neurological disorders, and future directions in the field of astrocyte heterogeneity.

## Introduction

**Figure 1:**
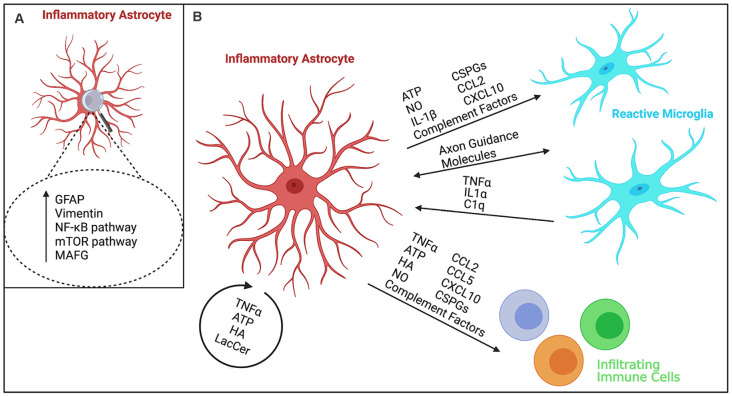
Schematic representation of inflammatory astrocytes and their interactions with other cells during neuroinflammation. **(A)** Markers upregulated in inflammatory astrocytes and pathways regulating their activation. While GFAP and Vimentin are commonly upregulated in reactive astrocytes (Sofroniew, 2009; Escartin et al., 2021), inflammatory astrocytes show increased expression of the NF-κB pathway (Zamanian et al., 2012; Lian et al., 2015) and activation of MAFG and mTOR signaling (Sofroniew, 2009; Zamanian et al., 2012; Li et al., 2015; Wheeler et al., 2020a). **(B)** Schematic overview of various known cytokine, chemokine, ionic, and protein interactions of inflammatory astrocytes with neighboring cells. Inflammatory astrocytes can affect microglia and infiltrating immune cells by secreting immune factors such as cytokines and chemokines (Sofroniew, 2009; Zamanian et al., 2012; Liddelow et al., 2017; Clark et al., 2019), complement proteins (Zamanian et al., 2012; Lian et al., 2016; Liddelow et al., 2017), as well as extracellular matrix molecules such as hyaluronan (HA; Kuipers et al., 2016; Nagy et al., 2019) and chondroitin sulfate proteoglycans (CSPGs; Keough et al., 2016; Stephenson et al., 2018), and cytotoxic factors, such as nitric oxide (NO), adenosine triphosphate (ATP; Orellana et al., 2011), and mitochondrial fragments (Joshi et al., 2019). These cells can, in turn, affect astrocyte reactivity as well (Colombo and Farina, 2016; Rothhammer et al., 2016; Liddelow et al., 2017; Williams et al., 2020; Clark et al., 2021). In particular, microglia have been shown to affect inflammatory astrocyte function (Liddelow et al., 2017; Yun et al., 2018; Joshi et al., 2019), while concurrently inflammatory astrocytes release many microglia-activating factors (Zamanian et al., 2012; Guedes et al., 2018) resulting in a feed-forward loop of activation. In addition, microglia-astrocyte crosstalk (Matejuk and Ransohoff, 2020) has been implicated in driving disease pathology, for example through the release of chemokines/cytokines (Itoh et al., 2017) and direct protein-protein interaction through axon guidance molecules, such as Sema4D/PlexinB2 and EphrinB3/EphB3 (Clark et al., 2021). Astrocytes can also activate themselves in an autocrine manner through the release of cytokines (Sofroniew, 2009; Zamanian et al., 2012; Escartin et al., 2021), ATP (Sofroniew, 2009; Zamanian et al., 2012), inflammatory HA (Kuipers et al., 2016; Nagy et al., 2019), and certain glycolipids such as lactosylceramide (LacCer; Mayo et al., 2014).

Astrocytes are the most abundant glial (non-neuronal) cell type of the central nervous system (CNS). Among many functions, they play a critical role in maintaining blood-brain barrier function (Engelhardt, 2003), supporting neurons and other glia (Jessen, 2004), and reacting to changes in both the local (Henrik Heiland et al., 2019; Shigetomi et al., 2019) and external environment (Rothhammer et al., 2016; Wheeler et al., 2020b). Beyond these homeostatic functions, astrocytes can respond to several stimuli and subsequently display profound genetic, morphological, and functional changes in a process termed reactive astrogliosis (Sofroniew, 2009; Escartin et al., 2021; Figure 1). Reactive astrogliosis can be triggered by injury (Cotrina et al., 2015; Okada et al., 2018), inflammation (Hansson et al., 2016), or stress (Chen et al., 2020; Kogel et al., 2021), and can result in a feed-forward process, where an initial stimulus induces a reactive astrocyte response, which triggers the release of intracellular and soluble factors that further drive this response. Reactive astrogliosis, reactive astrocyte response, and (astro)gliosis are terms often used interchangeably in the field of astrocyte biology. While their exact definition might differ between studies, potentially due to the heterogeneity in reactive astrocyte phenotypes and functions (see below), they generally refer to astrocyte responses to stimuli beyond physiological functions, as described above.

While the concept of (reactive) astrogliosis has been known since it was first observed early in the history of neuroscience (Rindfleisch, 1863; Müller, 1904), the heterogeneity of astrocyte phenotypes, function, and reactivity are becoming increasingly appreciated (Escartin et al., 2021). Due to this heterogeneity, the role of reactive astrogliosis in disease processes, such as neuroinflammation, is often multifaceted and remains an active area of research in the field. For example, reactive astrocytes can contribute to inflammation by promoting immune responses, but can also suppress these responses (Cordiglieri and Farina, 2010).

In parallel, at least two distinct types of reactive astrocytes were identified in initial studies examining the heterogeneity of astrocyte responses—inflammatory/neurotoxic and neuroprotective astrocytes, originally referred to as “A1” and “A2” astrocytes—analogous to proinflammatory M1 and anti-inflammatory M2 macrophages.

Inflammatory “A1” astrocytes are a classification of reactive astrocytes that are characterized by their neurotoxic, proinflammatory phenotype (Liddelow et al., 2017). They were first defined, alongside their neuroprotective counterparts, “A2” astrocytes, through pioneering experiments conducted in the Barres lab (Zamanian et al., 2012). To assess whether reactive astrocyte responses differ based on the insult given, they analyzed differentially expressed genes in reactive astrocytes that were induced either by experimental ischemic stroke or by neuroinflammation [through systemic administration of lipopolysaccharide (LPS; Zamanian et al., 2012)] and subsequently defined the two distinct activation states described above. However, similar to the evolution of the concept of M1/M2 macrophages, which has been expanded into a more continuous and plastic activation model, recent advances in single cell RNA sequencing (Wheeler et al., 2020a), as well as further analysis of the regional (Itoh et al., 2017; Boisvert et al., 2018; Williams et al., 2020) and phenotypic (Wheeler et al., 2020a) diversity of astrocytes, have made it apparent that the heterogeneity of (reactive) astrocytes extends beyond these two distinct states. In fact, a recent consensus review clarifying various idiosyncrasies in the field of astrocyte biology highlights the need to abandon the limited categorization of A1/A2 astrocytes, as the understanding of distinct astrocyte states has evolved beyond a binary paradigm (Escartin et al., 2021). Instead, a spectrum of reactive astrocyte states, characterized by gene expression signatures, as well as functional features, more accurately reflects astrocyte responses in neuropathology. Nevertheless, it is clear that under certain pathological conditions, astrocytes can adopt distinct inflammatory features and markers for this inflammatory phenotype are becoming more refined. Therefore, in this review, we will focus on the current state of literature on the role of inflammatory astrocytes in various neurological disorders.

Genes that are differentially upregulated in inflammatory astrocytes (the originally coined “A1” astrocytes) have been identified as critical players in various proinflammatory pathways, including the antigen presentation pathway, the complement pathway, and the interferon response pathway (Zamanian et al., 2012). Activation of the complement pathway can result in detrimental neuroinflammation (Lian et al., 2015; Okrój and Potempa, 2019), and complement component 3 (C3) is markedly enriched in inflammatory astrocytes compared to resting and neuroprotective astrocytes. Therefore, it is now frequently used in histology to identify inflammatory astrocytes, along with the upregulation of general reactive astrocyte markers, such as glial fibrillary acidic protein (GFAP; Escartin et al., 2021; Table 1). In addition, a common method to identify inflammatory astrocytes *in vitro* or *ex vivo* is to assess the expression of a set of genes that were found to be uniquely upregulated in the originally defined “A1” astrocytes (Liddelow et al., 2017), by quantitative PCR (Table 1). Because there is not one specific marker for this subtype and inflammatory genes can be expressed by other cell types (or even other astrocyte subpopulations) as well, particularly under neuroinflammatory conditions, a combination of markers should be used to properly determine the inflammatory phenotype of reactive astrocytes and rule out contamination of other cell types. In addition, functional features should also be taken into account when defining whether a particular astrocyte subtype observed in a neuropathological condition has inflammatory capacities.

**Table 1:** Commonly used techniques to identify inflammatory astrocytes.

Experimental Sample	Technique	Targets*
Tissue/cell culture	Immunohisto- or cytochemistry	Pan-reactive proteins: GFAP, Vimentin, S100β
		Inflammatory proteins: C3, GBP2
Cultured or sorted cells/tissue homogenates	qRT-PCR	Pan-reactive transcripts: Lcn2, Steap4, Serpina3n, S1pr3, Cxcl10, Hsbp1, Timp1, Aspg, Osmr, Cp, Vim, Gfap
		Inflammatory transcripts: C3, H2-D1, Serping1, H2-T32, Ggta1, Iigp1, Gbp2, Fbln5, Fkbp5, Srgn, Amigo2
Cultured/isolated cells/tissue	*In situ* hybridization	Pan-reactive probes: Lcn2, Serpina3n, Slc1a3
		Inflammatory probes: C3, H2-D1, Serping1

*For all detection methods a combination of one or more pan-reactive astrocyte markers, as well as inflammatory-specific markers, is used to confirm the inflammatory phenotype. Sources that contain specific information on antibodies, primer, and probe sequences include: Zamanian et al. (2012), Liddelow et al. (2017), Clarke et al. (2018), and Hartmann et al. (2019).

After their first genomic identification, subsequent studies have shown that *in vitro*, inflammatory astrocytes lose many of the homeostatic functions that astrocytes are known for, such as providing neurotrophic support, promoting synaptogenesis, and phagocytosis of synapses (Liddelow et al., 2017). It was shown that inflammatory astrocytes could be induced by soluble factors secreted by LPS-stimulated microglia. Of these factors, IL-1α, TNFα, and C1q, most potently in combination, were shown to be sufficient and necessary to polarize astrocytes to an inflammatory phenotype (Liddelow et al., 2017). Similarly, culturing naïve astrocytes with microglia conditioned media from Amyotrophic Lateral Sclerosis (ALS; Joshi et al., 2019) or Alzheimer’s Disease (AD; Xu et al., 2018) models resulted in these astrocytes taking on an inflammatory phenotype.

Inflammatory astrocytes have been the primary focus of neurological disease research, in part because techniques to identify neuroprotective astrocytes have remained elusive, whereas inflammatory astrocytes are more readily identified using the methods described above. As such, the potential role of inflammatory/neurotoxic astrocytes in neurodegenerative and neuroinflammatory diseases has recently been the subject of an increasing number of studies. Here, we discuss the roles that inflammatory astrocytes (may) play in these diseases, the efforts that are being made to pharmacologically target inflammatory astrocytes, and the limitations in studying this specific phenotype.

## Alzheimer’s Disease

Alzheimer’s disease is a progressive, neurodegenerative disease characterized by the accumulation of amyloid-beta plaques and neurofibrillary tangles of the microtubule-associated protein tau (Dickson and Vickers, 2001). The exact role of astrocytes in the propagation (or “seeding”) of tau tangles is debated and is a growing, active area of research. Astrocytes have been observed to internalize tau. However, it is yet unclear whether (or when) this internalization leads to degradation or propagation of tau, and whether this contributes to the induction of an inflammatory phenotype in astrocytes (Kovacs, 2020; Reid et al., 2020; Fleeman and Proctor, 2021). In AD, the presence of reactive astrocytes often precedes the formation of the disease’s characteristic histopathologies (Heneka et al., 2005; Orre et al., 2014). Moreover, a recent single cell analysis of non-neuronal cell populations in the 5xFAD transgenic mouse model of AD revealed a transient astrocyte response as the disease progresses, from a GFAP-low state to a GFAP-high state, as well as an AD-specific population termed “disease-associated astrocytes” (Habib et al., 2020). As such, there is great interest in determining the role of these reactive astrocytes in the pathogenesis of AD, and advances in RNA sequencing technology drive increasingly refined analyses of their phenotypes and functions.

To quantify inflammatory astrocyte responses, the density of C3^+^ cells with astrocyte morphology was analyzed in post-mortem AD tissue. C3^+^ astrocyte-like cells were found to be enriched in the upper cerebral cortex of patients. Interestingly, control tissue also showed significant numbers of C3^+^ astrocyte-like cells, notably in the lower cerebral cortex and white matter (King et al., 2020). In another study, AD subjects had significantly more C3^+^ reactive astrocytes compared to matched controls in the entorhinal cortex, one of the first brain regions affected in AD, and the hippocampus (Balu et al., 2019). The majority of these C3^+^ astrocytes also co-expressed serine racemase (SR); however, the density of these astrocytes was concentrated primarily in superficial rather than deep layers. SR is an enzyme that is critical for the conversion of L-serine to D-serine, which can bind to NMDA receptors. These human results were confirmed with a murine model of AD using aged TgF344-AD rats and it was additionally found that these rats had increased activation of signaling pathways associated with extrasynaptic NMDAR activation in the hippocampus (Balu et al., 2019). As inflammatory astrocytes have been shown to lose normal astrocyte functions (Liddelow et al., 2017) and extrasynaptic NMDAR activation is linked to the deleterious effects of glutamate on plasticity and neuronal survival (Bading, 2017), these results implicate a potential involvement of inflammatory astrocytes in the progression of AD. Indeed, in a murine tauopathy model, astrocytes were shown to display an inflammatory expression profile in the early stages of neurodegeneration. In addition, C3 immunoreactivity was confined to reactive astrocytes and genetic deletion of C3 resulted in reduced neuronal loss, suggesting that these inflammatory astrocytes might contribute to tau-driven pathology (Wu et al., 2019). In another murine AD model, activation of melanocortin receptors by its agonist D-Tyrosine resulted in a significant decrease in GFAP^+^/C3^+^ astrocytes in the CA1 region of the hippocampus (Lau et al., 2021). This decrease in inflammatory astrocyte numbers correlated with a significant decrease in amyloid plaques deposition and critical levels of toxic amyloid-β isomers in the hippocampus (Lau et al., 2021). These findings suggest that targeting GFAP^+^/C3^+^ astrocytes might be a potential therapeutic avenue in the treatment of AD. In addition, another study showed that *in vitro*, inflammatory astrocyte induction can be blocked by exogenously applied milk fat globule epidermal growth factor 8 (MFG-E8), production of which is reduced in these inflammatory astrocytes (Xu et al., 2018). In a study highlighting the glial effects of amyloid-β exposure in an AD model, activation of the NF-κB pathway, known to be involved in inflammatory astrocyte induction, was detected in astrocytes and subsequent neuronal release of C3 resulted in synaptic dysfunction (Lian et al., 2015). These studies highlight potential pathways to modulate inflammatory astrocyte activation and improve AD pathology.

Cerebral amyloid angiopathy (CAA) is a typical condition of AD pathology and is characterized by cerebrovascular deposition of amyloid protein. While the function of the amyloid protein remains elusive, its accumulation is toxic and known to induce apoptosis and drive neurodegeneration (Chow et al., 2010; Chen et al., 2017). In a murine model of early CAA, immune and glial responses were analyzed, and histology revealed perivascular reactive astrogliosis, identified by GFAP (reactive astrocytes), Thioflavin-S (vascular amyloid), and α-smooth muscle actin (vascular smooth muscle cells) immunoreactivity, in 9-month-old mice. Of note, this phenomenon was absent in 3-month-old mice, suggesting a temporal, progressive astrocyte response. Further characterization of this response revealed a robust inflammatory astrocyte presence, as defined by colocalization of C3 and GFAP, in the hippocampus and cerebellum (Taylor et al., 2020).

As the accumulation of plaques and tangles occurs before the onset of symptoms, and there is a significant benefit of early intervention to patients, there is a strong drive to establish biomarkers for early AD pathology, as well as to develop non-invasive treatments. In this regard, the retina has become a popular area of study given its common embryological origin with the brain (Paquet et al., 2007). In the pursuit of an early biomarker for AD, upregulation of IL-1β in microglia and the additional presence of inflammatory astrocytes, as determined by GFAP and C3 colocalization, was found in retinal tissue from AD patients, indicating that the inflammatory activation of astrocytes is a feature of early AD pathology (Grimaldi et al., 2019). In addition, in a study comparing astrocyte-derived exosomes (ADE) in the plasma of AD patients to those of matched controls, levels of complement proteins and cytokines were analyzed (Goetzl et al., 2019). Complement factors, including C3d and C1q, one of the factors able to induce inflammatory astrocytes *in vitro*, were significantly higher in ADE from AD patients. With respect to cytokine profiles, while there was greater overlap between the two groups, AD ADE contained higher levels of IL-6, TNF-α and IL-1β (Goetzl et al., 2019), cytokines known to be involved in reactive astrogliosis (Choi et al., 2014).

Moreover, exercise is thought to be of benefit as a treatment for AD due to its capacity to stimulate the release of neurotrophic factors (Prado Lima et al., 2018), decreasing deposition of amyloid-β plaques (Prado Lima et al., 2018), and improving tau pathology (Belarbi et al., 2011; Fleeman and Proctor, 2021). In a study using exercise to treat a murine model of AD, rotarod exercise therapy resulted in a decrease of inflammatory astrocytes, along with reduced amyloid-β deposition, neuronal loss, and cognitive decline, showing that astrocyte reactivity correlates with treatment effects as well (Nakanishia et al., 2021).

## Huntington’s Disease

Huntington’s disease (HD) is a neurodegenerative disease, primarily affecting the basal ganglia, that is caused by a dominantly inherited CAG trinucleotide repeat expansion in the huntingtin gene on chromosome 4 (McColgan and Tabrizi, 2018). In humans, reactive (fibrillary) astrogliosis within the corpus striatum is used to classify progressive stages of HD (Rüb et al., 2016). It has been shown that astrocytes from HD patients become physiologically and morphologically activated when exposed to mutant huntingtin, as determined by increased GFAP staining and morphological changes—specifically, thicker processes and a larger somata (Faideau et al., 2010). Additionally, these reactive astrocytes have significantly decreased expression of the glutamate transporters GLAST and GLT-1, which leads to a subsequent decrease in a critical astrocyte function—glutamate uptake (Rose et al., 2018). This is of interest, as loss of physiological astrocyte functions is characteristic of *in vitro* generated inflammatory astrocytes (Liddelow et al., 2017).

Single-nucleus RNA sequencing of astrocytes derived from the postmortem anterior cingulate cortex of HD and control human tissue went beyond the “A1/A2” classification and identified several distinct astrocyte “states” as determined by differential gene pattern expression (Al-Dalahmah et al., 2020). Additionally, this study confirmed that astrocytes in the caudate nucleus of HD grades III and IV express markers of an inflammatory state, showing C3 staining alone and double immunostaining for C3 and GFAP (Al-Dalahmah et al., 2020). These results suggest that inflammatory astrocytes in the anterior cingulate cortex are associated with progressive stages of HD.

Genomic and proteomic analysis of striatal astrocytes shows only the inflammatory astrocyte-associated gene *Serping1* to be consistently upregulated across human samples and murine models (Diaz-Castro et al., 2019). However, akin to what has been reported in previous inflammatory astrocyte literature (Liddelow and Barres, 2017), astrocytes from HD striatum undergo significant morphological and transcriptional changes. Moreover, these changes are largely reversed by lowering mutant Huntington protein specifically in astrocytes (Diaz-Castro et al., 2019) showing a direct effect of mutant protein on reactive astrogliosis and highlighting the potential for therapeutics targeting reactive astrocytes in HD.

## Multiple Sclerosis

Multiple Sclerosis (MS) is a progressive autoimmune demyelinating disease, characterized by infiltration of peripheral immune cells that target myelin within the CNS, and resulting in focal neuroinflammatory lesions, demyelination, and neurodegeneration. Astrocytes are thought to be involved in MS pathogenesis due to their capacity to promote entry of peripheral immune cells to the CNS, as well as to directly affect inflammatory processes in lesion formation (Ponath et al., 2018). One of the most widely used animal models used in MS research is experimental autoimmune encephalomyelitis (EAE), which involves inducing a T cell-driven immune response against myelin that leads to infiltration of these cells into the CNS, activation of resident cells, including astrocytes, and subsequent destruction of myelin, and damage to axons and neurons (Rangachari and Kuchroo, 2013; Lassmann and Bradl, 2017). As the model is driven by an immune response, EAE is often used to study the role of infiltrating and resident inflammatory cells in demyelination, because it recapitulates the inflammatory milieu found in actively demyelinating MS lesions (Lassmann and Bradl, 2017). In addition, MS is often studied in conjunction with optic neuritis, which is also pathologically characterized by peripheral immune cell infiltration (Bettelli et al., 2003; Lassmann and Bradl, 2017). Reactive astrocytes can be found at various stages of MS lesions. In addition to their abundance in chronic lesions, reactive astrocytes are present in the center and the active edge of acutely demyelinating lesions, as well as in bordering white matter (Kuhlmann et al., 2017; Ponath et al., 2017, 2018). In parallel, astrocytes become reactive, as determined by enhanced expression of GFAP, early and throughout EAE pathogenesis (Wang et al., 2005; Luo et al., 2008; Pham et al., 2009). C3 containing astrocytes are abundantly present in the center, as well as the expanding edge of actively demyelinating MS lesions, and can also be found in chronic lesion stages. Interestingly, these C3^+^ astrocytes are often located in close proximity to activated microglia/macrophages (Ingram et al., 2014; Liddelow et al., 2017).

In EAE, inflammatory astrocytes, as defined by C3 staining and inflammatory-specific transcript analysis, are prevalent in the retina and optic nerve tissue and are associated with retinal ganglion cell loss (Jin et al., 2019). Additionally, the complement cascade was found to be one of the most significantly upregulated pathways in the optic nerve of EAE mice (Tassoni et al., 2019). These results suggest that inflammatory astrocytes could be a potential target against some common visual symptoms of MS resulting from optic nerve degeneration. Additionally, this study observed significantly more C3 expressing astrocytes within the optic nerve of female mice as compared to males (Tassoni et al., 2019). This is of note, as the prevalence of MS is significantly higher in women than in men.

Recently, a pro-inflammatory and neurotoxic signature was also found in an astrocyte subset that is greatly expanded during EAE, identified by single cell RNA sequencing analysis. This subset is characterized by activation of the NF-κB and inducible nitric oxide synthase (iNOS) pathways, reduction of the NRF2 pathway, which limits oxidative stress and inflammation, and increased expression of the master transcriptional regulator MAFG (Wheeler et al., 2020a). Moreover, an astrocyte subset with similar features can be found in a combined scRNAseq dataset containing data from MS and control tissue samples. This inflammatory subset is detected in the majority of patient samples (12 out of 20) and greatly expanded in samples from MS patients compared to control samples (25-fold; Wheeler et al., 2020a).

There are various other models of MS that represent additional facets of its pathogenesis, such as the cuprizone model of demyelination and toxin-induced demyelination and remyelination (Lassmann and Bradl, 2017). However, studies exploring the presence and role of inflammatory astrocytes in these models are very limited.

One of the only known factors to correlate with MS progression is age, the majority of MS patients developing a progressive stage of the disease when they are between 40–50 years old (Tremlett and Zhao, 2017). This is significant, as both immune function and astrocyte functions (Palmer and Ousman, 2018), such as morphological changes (Jyothi et al., 2015), increased GFAP expression (Wu et al., 2005; Clarke et al., 2018), and activation of complement factors (Clarke et al., 2018), are known to change over time. In particular, aging astrocytes take on a more inflammatory phenotype (Clarke et al., 2018). In a study of over 1,000 proteins derived from the cerebrospinal fluid of 431 patients, a cluster of inflammatory astrocyte-derived proteins was found to be significantly upregulated in MS patients and had a significant, reproducible correlation with MS severity (Masvekar et al., 2019). Together, these findings suggest that inflammatory astrocytes may play an active role in various stages of MS pathogenesis and could provide a target for addressing damage to the optic nerve, as well as the CNS parenchyma of MS patients. Moreover, proteins derived from inflammatory astrocytes could prove to be a valuable biomarker to predict the progression of disease in MS.

## Parkinson’s Disease

Parkinson’s disease (PD) is a progressive neurodegenerative disorder characterized by loss or degeneration of dopaminergic neurons in the substantia nigra in the midbrain and the development of Lewy bodies, protein aggregates that primarily contain the protein α-synuclein (Forno et al., 1986; Braak et al., 2003). It is a disease of unknown etiology commonly associated with aging and family history (Kalia and Lang, 2015). As a result of neurodegeneration, inflammation also plays a key role in PD. When neurons die, they release proinflammatory and cytotoxic molecules (Glass et al., 2010) that promote gliosis and immune responses. These responses lead to a feed-forward cycle wherein activated immune cells further respond by releasing additional proinflammatory factors (Lee et al., 2019), thereby perpetuating inflammation and neuronal damage.

The key contributors to PD pathogenesis with a proinflammatory relationship are astrocytes and microglia. A widely used model for PD is the MPTP model, based on the toxic properties of peripherally administered 1-methyl-4-phenyl-1,2,3,6-tetrahydropyridine (MPTP), which results in dopaminergic neurodegeneration in the striatum and substantia nigra, a pattern similar to the human disease (Meredith and Rademacher, 2011). In this model, systemic administration of LPS exacerbates microglial activation and induces the conversion of astrocytes to an inflammatory (C3^+^) phenotype (García-Domínguez et al., 2018). This shows that peripheral inflammation can trigger inflammatory astrocyte conversion under the conditions of dopaminergic neurodegeneration (García-Domínguez et al., 2018). In another murine model of PD, in which LPS is injected into the midbrain, genes associated with inflammatory astrocytes, as well as the potassium channel subunit Kir6.2, were upregulated in the substantia nigra (Song et al., 2021). Kir6.2 is induced by chronic metabolic stress, associated with the degeneration of dopaminergic neurons and can act as an inflammatory mediator (Liss et al., 2005; Du et al., 2014). Kir6.2 was shown to be expressed by astrocytes and its genetic deletion mitigated inflammatory astrocyte expression patterns and prevented dopaminergic neurodegeneration and behavioral deficits (Song et al., 2021). Additionally, it has been shown *in vivo* that NLY01, a glucagon-peptide-1 receptor agonist, is capable of blocking the astrocytic conversion to an inflammatory phenotype by preventing the microglial release of IL-1α, TNFα, and C1q (Yun et al., 2018). NLY01 was shown to be protective in two models of PD: the α-synuclein preformed fibrils (PFF) model of sporadic PD, and the progressive, lethal, constitutive α-synucleinopathy model (Yun et al., 2018). Furthermore, *β*-sitosterol-*β*-D-glucoside (BSSG) is a neurotoxin found in cyad seeds and its chronic consumption induces a progressive PD-like disease in humans and rats (Van Kampen and Robertson, 2017). In a study assessing the neuroinflammatory reaction to this neurotoxin, a significant induction of inflammatory astrocytes, verified by co-staining of C3 with GFAP or S100β, was observed with a single BSSG injection to the substantia nigra, correlating with loss of dopaminergic neurons (Luna-Herrera et al., 2020). Together, these studies illustrate the involvement of inflammatory astrocytes in the pathogenesis of PD and provide potential targets to regulate their induction.

## Prion Diseases

Prion diseases are a group of neurodegenerative diseases caused by the conversion of prion protein (PrP) to an abnormal, misfolded form of the protein (PrP^Sc^). This conversion is characterized by a shift of the normal prion protein α-helical structure to a β-pleated sheet structure, which forms amyloid deposits. The shift of PrP into PrP^Sc^ in prion diseases has a cascading effect, where the misfolded PrP^Sc^ protein acts as a seed and propagates the misfolding of additional proteins. However, the mechanism for this cascade is unknown. Among neurodegenerative disorders, prion diseases are unique because they can occur either spontaneously, genetically, or by transmission. The most common prion diseases are Creutzfeldt-Jakob Disease (CJD), its bovine equivalent Bovine Spongiform Encephalopathy (BSE, “mad cow disease”), and scrapie in sheep and goats (Belay, 1999; Geschwind, 2015).

Reactive astrogliosis is a hallmark of all prion diseases. Astrocytes play roles in prion diseases both in their capacity as proponents of neuroinflammatory response and as promotors of PrP^Sc^ spread and aggregation (Carroll and Chesebro, 2019). In a murine model of prion disease, induced *via* intracerebral injection of scrapie brain homogenate, reactive astrogliosis occurs early and throughout the clinical course and coincides with PrP^Sc^ deposition (Tribouillard-Tanvier et al., 2012). While prion diseases were originally thought to involve a limited neuroimmune response (Belay, 1999; Geschwind, 2015), analysis of cytokines and chemokines in the scrapie inoculation-induced mouse model showed that protein levels of, among many others, IL-1Ra, CXCL10 (IP-10), and CCL5 (RANTES) were significantly increased as the disease progressed (Carroll et al., 2015). These factors are also among many produced by *in vitro* activated human astrocytes (Choi et al., 2014). Additionally, the majority of the inflammatory genes upregulated in scrapie-inoculated mice can be induced by the NF-κB pathway, which is activated in these mice (Tribouillard-Tanvier et al., 2012; Carroll et al., 2015). This is of note as NF-κB pathway activation has been associated with C3 production by astrocytes as well (Lian et al., 2015).

Numerous inflammatory (C3^+^ or GBP2^+^) astrocytes can be found in tissues of both murine prion disease and human CJD cases (Hartmann et al., 2019), and expression of C3 and GBP2 is significantly upregulated in CJD brain tissue and is associated with disease duration and risk genotype (Ugalde et al., 2020). Blocking the induction of inflammatory astrocytes in prion-infected triple KO mouse (TKO-mice), lacking TNFα, IL-1α, and C1q expression, however, does not affect PrP^Sc^ protein titers or deposition. Moreover, the disease course is significantly accelerated in these mice, indicating that the inflammatory response of astrocytes might constitute a protective mechanism limiting the damaging effects of PrP^Sc^ accumulation (Hartmann et al., 2019). While their presence and involvement in the pathogenesis of prion diseases is apparent, the overall effect of their involvement, whether helpful or harmful, remains to be seen.

## Discussion

Inflammatory astrocytes have been shown to play roles in inflammatory and neurodegenerative mechanisms of neurological disease. Of note, in the healthy aging brain, astrocytes have also been shown, *in vivo*, to take on a genetic profile similar to that seen in neuroinflammation-induced astrocytes (Clarke et al., 2018). Aging is a significant risk factor for many CNS pathologies (Palmer and Ousman, 2018; Hou et al., 2019); for example, it correlates with disease progression in MS (Confavreux and Vukusic, 2006; Koch et al., 2009, 2010; Hou et al., 2019). It is therefore critical to explore the role of these aging and potentially inflammatory astrocytes in the exacerbation of disease and injury. It is likely that aging-induced inflammatory astrocytes contribute to neuroinflammation and neurodegenerative processes in general, given the demonstrated neurotoxic functions of inflammatory astrocytes (Liddelow et al., 2017), their prevalence with age (Clarke et al., 2018), and their observed presence in many neurodegenerative diseases (Liddelow and Barres, 2017; Clarke et al., 2018; Goetzl et al., 2019; Hartmann et al., 2019; Tassoni et al., 2019; Song et al., 2021).

A confounding factor in all studies examining the role of astrocytes in disease pathogenesis is the fact that astrocyte and microglial activation commonly happens in concert. Due to obvious experimental challenges, many of the studies discussed in this review do not directly address the potential inflammatory feed forward cycle of inflammatory or generally reactive astrocytes and activated microglia that might contribute to the progression of disease (Figure 1). Inflammatory activation of astrocytes can result in the release of proinflammatory cytokines that activate microglia and mediate neurotoxic inflammation. In turn, the proinflammatory factors released by activated microglia can further activate inflammatory astrocytes (Liddelow et al., 2017), thereby creating a detrimental, inflammatory feed forward cycle that exacerbates disease severity. Therefore, disease-modifying treatments targeting inflammatory astrocytes are of great interest, as eliminating a main component of this inflammatory cycle can mitigate its damage.

While still an area to be further explored, there have been some advances in therapeutically targeting inflammatory astrocytes, specifically, during disease (Table 2). NLY01, used to block the induction of inflammatory astrocytes by inhibiting the release of IL-1α, TNF-α and C1q from microglia, was successfully used in studies of PD (Yun et al., 2018) and glaucoma-associated neurodegeneration (Sterling et al., 2020) to ameliorate disease severity. With respect to ALS, studies regarding the transplantation of glial precursor cells demonstrated glial transplantation as a method to delay disease onset and ameliorate clinical symptoms (Kondo et al., 2014; Izrael et al., 2020). This has led to a current clinical study (National Library of Medicine, NCT03482050, 2018) of intrathecal transplantation of human-grade astrocytes in the hopes of reducing the large population of inflammatory astrocytes causing damage in patients with ALS (Izrael et al., 2020). These treatments show the potential of this avenue in disease management.

**Table 2:** Therapeutic and mechanistic methods of targeting inflammatory astrocytes.

Disease	Method	Primary target	Model system
Alzheimer’s Disease	Activation of melanocortin receptor by D-Tyrosine (Lau et al., 2021).	Astrocytes	*in vivo*
Alzheimer’s Disease	Exercise (Belarbi et al., 2011; Nakanishia et al., 2021).	Astrocytes	*in vivo*
Amyloid Lateral Sclerosis	Intrathecal transplantation of human-grade astrocytes (Izrael et al., 2020).	Astrocytes	*in vivo* (mouse and human)
Glaucoma Associated Neurodegeneration	Preventing microglial release of IL-1α, TNFα, and C1q by NLY01 (Sterling et al., 2020).	Microglia	*in vivo*
Huntington’s Disease	Transcriptional repression of mutant huntingtin protein using zinc finger proteins (Diaz-Castro et al., 2019).	Astrocytes	*in vivo*
Parkinson’s Disease	Dopamine D2 receptor agonist inhibition of NLRP3 inflammasome activation in astrocytes (Zhu et al., 2018).	Astrocytes	*in vivo*
Parkinson’s Disease	Genetic deletion of Kir6.2 (Song et al., 2021).	Astrocytes	*in vivo*
Parkinson’s Disease	Prevent microglial release of IL-1α, TNFα, and C1q by NLY01 (Yun et al., 2018).	Microglia	*in vitro*/*in vivo*
Prion Disease	Genetic deletion of TNFα, IL-1α, and C1q triple KO (Hartmann et al., 2019).	Microglia	*in vivo*
Multiple Sclerosis	NLRP3 inflammasome inhibition (Hou et al., 2020).	Astrocytes	*in vivo*

There are significant limitations in studying inflammatory astrocytes, as the mechanism underlying their induction has only been shown *in vitro* (Liddelow et al., 2017) and upregulation of genes associated with inflammatory astrocytes is not ubiquitous in all neurodegenerative diseases and their models. By extension, identifying the inflammatory nature of reactive astrocytes in particular settings is challenging. However, the previously mentioned recent consensus article provides clarity regarding markers and terminology to be used when describing reactive astrocytes (Escartin et al., 2021). Thus far, the methods used to identify inflammatory astrocytes are generally two-fold: co-expression of GFAP and C3 along with a transcription of a commonly defined subset of inflammatory astrocyte-specific genes. However, C3 upregulation is not unique to inflammatory astrocytes, as the complement cascade is activated in numerous inflammatory conditions (Markiewski and Lambris, 2007). Therefore, co-expression must be clearly shown and verified—preferably through quantitative techniques. Often these methods of identification are used individually or in conjunction to identify the presence of inflammatory astrocytes; however, studies that go beyond this correlation and delve into the mechanisms of inflammatory astrocyte induction and its consequences are still limited.

An additional challenge in determining the contribution of inflammatory astrocytes to neurological disorders is the innate limitations of using animal models. While rodent models provide valuable tools to dissect biological processes, there are various physiological differences between rodents and humans that need to be taken into account when extrapolating findings (Perlman, 2016). With regard to astrocyte responses, while general (reactive) gene expression profiles are similar between human and mouse astrocytes, differences in the molecular pathways induced by some stimuli do exist and it cannot be ruled out that the distinct expression profiles and functions of human astrocytes differ from those in the mouse models used to determine their role in neuropathology (Li et al., 2021). In addition to animal models, the postmortem tissue of patients is a valuable source to determine disease-specific mechanisms. However, many factors that are difficult (or impossible) to control can introduce variation in data and confound findings, such as the cause of death, stage of disease at the time of death, and postmortem interval (time from death to autopsy; Di Lullo and Kriegstein, 2017). For example, available tissue from postmortem sources is generally skewed towards the end or advanced stage of the disease, whereas biopsy material is often from cases that display an abnormal disease pattern. Moreover, obtaining control tissue (either from healthy individuals or non-related neurological conditions) that is properly matched for sex, gender, age, and lifestyle factors is challenging. Therefore, using a combination of techniques, models, and tissue sources is best suited to dissect the intricate interplay of the cellular and molecular mechanisms driving pathology. Moreover, the exact function of specific reactive astrocyte states or subtypes likely depends on the pathological context and stage of disease, due to the suggested transient and/or plastic nature of reactive astrocyte states/subtypes (Mayo et al., 2014; Habib et al., 2020). Only once the exact contribution of inflammatory astrocytes to the various stages of the disease has been mapped, targeting this population specifically at the appropriate stage could provide an effective treatment strategy.

Developments in the field of single cell RNA sequencing have advanced studies of reactive astrocyte responses beyond the initial binary classification of inflammatory/neurotoxic and neuroprotective astrocytes. For example, several unique clusters of reactive astrocytes were identified in EAE and MS tissue (Wheeler et al., 2020a), and differential effects of ablation of reactive astrocytes at different stages of EAE suggest that this astrocyte response might be transient and/or plastic (Mayo et al., 2014). In addition, transient and disease-specific reactive astrocyte populations were observed in the 5xFAD model of AD (Habib et al., 2020). Therefore, as discussed before, a refined view of astrocyte heterogeneity and plasticity allows for a more comprehensive classification of reactive astrocyte populations/states and potentially a greater understanding of their role in disease pathology.

Recent additional advances have also allowed for exploration in the field of cell-cell crosstalk. In a recent study using an mRNA barcoding technique that takes advantage of the pseudorabies virus’s capacity to spread between interacting cells (coined RABID-seq), labeling cells interacting with astrocytes showed that pathogenic astrocytes connected to microglia display an inflammatory signature, and that their crosstalk is mediated, amongst others, by axon guidance molecules (Clark et al., 2021). Advances such as these are critical as they allow for an understanding of the complex cellular interactions that perpetuate inflammation. However, it also forebodes that, as the narrative of the involvement of astrocytes in disease continues to develop, it may be that the classification of astrocyte subsets will be defined more by their function in relation to a specific disease state, rather than a specific binary phenotype based on gene expression signatures.

## Author Contributions

JR performed literature searches, and structured and wrote the manuscript. HK gave structural and contextual input, and edited the manuscript. All authors contributed to the article and approved the submitted version.

## Conflict of Interest

The authors declare that the research was conducted in the absence of any commercial or financial relationships that could be construed as a potential conflict of interest.

## Publisher’s Note

All claims expressed in this article are solely those of the authors and do not necessarily represent those of their affiliated organizations, or those of the publisher, the editors and the reviewers. Any product that may be evaluated in this article, or claim that may be made by its manufacturer, is not guaranteed or endorsed by the publisher.
